# APDB: A Large-Scale Repository of Predicted 2D and 3D Aptamer Structures

**DOI:** 10.1021/acs.jcim.6c02031

**Published:** 2026-09-17

**Authors:** Kabelo P Mokgopa, Kevin A Lobb, Tendamudzimu Tshiwawa

**Affiliations:** † Department of Chemistry, 59100 Rhodes University, Makhanda 6140, South Africa; ‡ Research Unit in Bioinformatics (RUBi), Rhodes University, Makhanda 6140 South Africa

## Abstract

RNA aptamers are short, single-stranded oligonucleotides capable of folding into specific three-dimensional structures to bind diverse molecular targets with high affinity and specificity. In this study, we present a comprehensive computational analysis of a curated data set of over 150,000 RNA aptamers annotated with sequences, predicted secondary structures, molecular features, and minimum free energy (MFE) values. A total of 161 molecular descriptors were extracted, including pairing ratios, stem density, sequence complexity, and motif composition. Dimensionality reduction using Principal Component Analysis (PCA) and t-distributed Stochastic Neighbor Embedding (t-SNE) revealed distinct clustering patterns driven by MFE, GC/AU pairing composition, and secondary structure complexity. PCA indicated that aptamers with high AU content tend to occupy distinct structural space, while t-SNE highlighted concentrated regions of high GC pairing and stem density. A 3D surface plot of GC pairing, AU pairing, and MFE further demonstrated a nonlinear landscape, with folding stability changing in a bumpy, hill-like pattern as pairing ratios varied. These findings provide insights into how base-pairing composition influences aptamer folding stability and offer a framework for rational design. The resulting Aptamer Database (APDB) integrates these analyses into an interactive, scalable platform to support research in biosensing, therapeutics, and computational aptamer design (https://boron.ru.ac.za/database).

## Graphical abstract

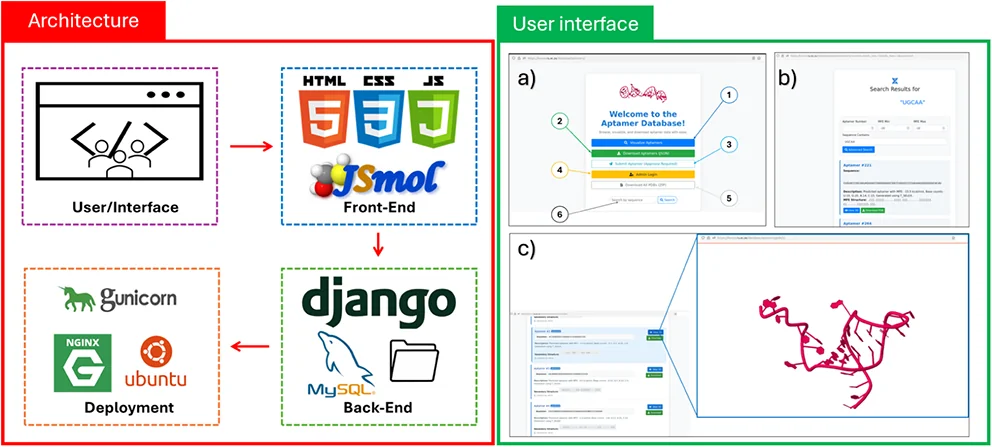

## 1 Introduction

In the rapidly evolving landscape of molecular biology and bioinformatics, the capacity to identify, characterize, and manipulate biomolecular interactions is central to advancing diagnostics, therapeutics, and biotechnology.^1–4^ Among the most versatile molecular recognition tools developed in recent decades, aptamers have gained attention as short, synthetic single-stranded oligonucleotides (RNA or DNA) that bind with high affinity and specificity to a wide range of biological and chemical targets.^5,6^ Through intricate secondary and tertiary folding, aptamers can mimic antibody behavior while offering unique advantages, including chemical stability, low immunogenicity, ease of synthesis, and reversible denaturation.^7–10^

Aptamers are primarily selected via SELEX (Systematic Evolution of Ligands by EXponential enrichment) an iterative in vitro process that isolates high-affinity binders from large combinatorial libraries.^11^ Since their development in the 1990s, aptamers have found wide application in biosensing, targeted drug delivery, biomarker discovery, cancer therapy, antiviral research, and environmental monitoring.^12–16^ Their synthetic and programmable nature situates them at the intersection of molecular engineering and precision medicine.^15^ Although SELEX remains a central approach for aptamer discovery, recent developments have increasingly expanded the field toward in-silico approaches for aptamer design, sequence selection, affinity prediction, and structural optimization. Computational and artificial-intelligence-based methods are now being explored as complementary strategies for reducing the search space and accelerating the identification of promising aptamer candidates.^16–18^ For example, generative models have been applied to explore aptamer sequence space, while artificial intelligence has been investigated for predicting aptamer–target affinity and guiding sequence selection.^16,17^ Recent publications and reviews further highlight the growing role of non-SELEX computational strategies in de novo aptamer design and analysis.^19,20^

As aptamer research and application accelerate, so too does the need for structured, accessible, and interoperable data resources. In molecular sciences, databases such as ChEMBL,^21^ PubChem,^22^ and ZINC^23^ have enabled researchers to investigate molecular interactions, bioactivity profiles, and pharmacokinetics across thousands of compounds through molecular modeling. In natural product research, resources like the South African Natural Compound Database (SANCDB),^24^ NPASS,^25^ and COCONUT^26^ facilitate compound discovery and chemical diversity analysis. Structural biology has similarly benefitted from databases including the Protein Data Bank (PDB),^27,28^ SCOP,^29^ CATH,^30^ and platforms like NAKB^31^ which contains curated 3D macromolecular structures.

Despite these advancements, aptamer-specific data infrastructure remains underdeveloped. Current databases such as Apta-Index,^32^ Aptamer base,^33^ UTexas Aptamer Database,^34^ and Ribocenter-aptamer^35^ primarily focus on linear sequences, target names, binding affinities, and limited experimental metadata. Few offer structural representations, and even fewer provide tools for integrated visualization or comparative analysis of aptamer conformational features, which are critical to understanding function, improving binding affinity, and supporting computational design. Furthermore, with regard to these databases, most of them have data for less than 5 000 aptamers, and further, very few of them have any data with respect to RNA.

The growing shift toward computational and AI-assisted aptamer discovery therefore creates an additional need for large and structurally annotated data sets.^16^ Computational approaches can explore sequence and structural spaces that are difficult to examine experimentally at comparable scale, but their effectiveness depends strongly on the availability of sufficiently large and informative data sets for model development, benchmarking, and hypothesis generation.^17^ In particular, integrating sequence information with predicted secondary structures, three-dimensional conformations, and thermodynamic descriptors provides a basis for investigating relationships between sequence composition, structural organization, and folding stability.

To address this gap, we introduce a new comprehensive resource: the Aptamer Database (APDB). APDB curates over 150,000 RNA random generated aptamer entries, each annotated with nucleotide sequences, predicted secondary structures, 3D molecular conformations, and functional metadata. The platform is designed not only to enable structural insight but also to integrate into drug discovery pipelines, machine learning models, and rational design workflows. While the current version centers on RNA aptamers, future iterations will incorporate DNA aptamers, aptamer–target complexes, and ligand-bound states to expand the database’s utility.

## 2 Materials and Methods

### 2.1 Data Creation and Collection

#### 2.1.1 Aptamer Sequence Generation

Aptamer sequences were generated using the Base Randomization Algorithm (BRA)^36^ implemented in the custom-built T_SELEX package.^37^ This algorithm was designed to produce a diverse library of synthetic RNA aptamer sequences, incorporating over 150,000 unique aptamers with varying nucleotide compositions and lengths. BRA allows fine-tuned control over base distributions and motifs, supporting the creation of sequences suitable for downstream folding and structural prediction.^36^

#### 2.1.2 Secondary Structure Prediction

All generated aptamer sequences were folded using the T_SELEX package, which integrates the RNAfold algorithm^38^ from the ViennaRNA suite.^39,40^ For each sequence, the minimum free energy (MFE) structure was computed, and dot-bracket or pseudoknot notations that represent secondary structural motifs were recorded.^38^ The algorithm accounts for pseudoknots, base-pairing probabilities, and thermodynamic stability to generate biologically plausible secondary structures.

#### 2.1.3 3D Structure Prediction

The predicted secondary structures were subsequently passed through a T_SELEX module that interfaces with the RNAComposer^41^ web server to generate three-dimensional (3D) RNA structures. The dot-bracket outputs from RNAfold^38^ were submitted as input to RNAComposer^41^ via an automated pipeline that supports batch processing and structure annotation. For each aptamer, the Model 1 structure generated by RNAComposer^37^ was selected for inclusion in APDB. Model 1 represents the refined and minimized structure produced by the RNAComposer modeling procedure. During model generation, RNAComposer identifies and assembles appropriate structural elements from experimentally determined RNA structures, followed by structural refinement through minimization in torsion-angle space and subsequent minimization in atomic Cartesian-coordinate space.^42^ Thus, the 3D structures deposited in APDB correspond to the refined and minimized Model 1 generated by RNAComposer rather than an unrefined initial structural assembly.

### 2.2 APDB Architecture

#### 2.2.1 Data Storage and Web Server Implementation

The web server was developed using the Django framework^43^ and powered by a MySQL^44^ relational database, offering a robust, scalable backend for managing aptamer records, user interactions, and structural data. All aptamer-related information is organized through the custom-built AptamerData model, which serves as the central data structure within our system. Each aptamer entry is uniquely identified by an autoincrementing primary key and stores the full RNA sequence alongside detailed nucleotide composition, including counts of adenine (A), cytosine (C), guanine (G), and uracil (U). Structural information is also recorded, such as the minimum free energy (MFE) value and its associated secondary structure in dot-bracket notation. Tertiary structure data is referenced via file paths linking to PDB files generated by RNAComposer,^41^ and each entry also contains metadata about the uploader, submission time, data type, and an optional description.

In addition to core aptamer records, the platform tracks user activity and engagement through a dedicated ActivityLog model that logs visit, upload, and download actions, along with timestamps and optional annotations. For interactive functionality, such as user inquiries or support requests, a Message model is included, allowing authenticated users to post messages that are stored with sender identity and timestamp metadata.

To support community contributions and ensure quality control, the AptamerSubmission model handles a separate submission workflow. This model allows users to submit new aptamer sequences along with optional secondary and tertiary structural data. Each submission is timestamped, linked to the uploader’s contact information, and includes a review mechanism where entries can be approved or rejected by an administrator, with rejection reasons documented for feedback purposes. Submitted 3D structures are stored in a parallel directory structure and remain separate from the main data set until reviewed.

The database schema is intentionally designed to decouple raw data from user submissions, enabling data integrity, versioning, and future expansion. Django’s ORM (Object Relational Mapper)^45^ is used selectively, with managed tables disabled for core models to maintain compatibility with preexisting or externally managed databases. The server also supports secure file handling, dynamic data queries, and scalable interaction with the aptamer data set through Django views and templates.

### 2.3 Web Interface and Front-End Technologies

The front-end of the aptamer web server was developed using standard HTML5 and JavaScript, styled with the Bootstrap 4.5 framework to ensure a responsive and user-friendly design across devices. The interface supports both functional browsing and detailed structural visualization of aptamers. All dynamic content is rendered using Django’s template engine, enabling seamless integration between database content and web presentation. The platform includes dedicated HTML templates for various interactions such as aptamer submission, search results, and structure visualization.

APDB integrates VARNA^46^ to visualize predicted RNA secondary structures, providing users with an intuitive representation of the folding architecture of individual aptamers. A key feature of the front-end is the interactive 3D visualization of RNA tertiary structures, implemented using the JSmol^47^ JavaScript library. PDB files generated through RNAComposer^41^ are embedded and rendered within the web page, allowing users to rotate, zoom, and explore aptamer structures in real time. Additional interactive controls, such as toggling molecular spin or visual style (e.g., cartoon, wireframe), are implemented with JavaScript functions directly linked to JSmol^47^ scripting commands.

Templates such as view_pdb.html provide a detailed layout that combines molecular visualization with contextual metadata, including the aptamer sequence, secondary structure in dot-bracket notation, minimum free energy, uploader information, and structural descriptors. All pages are styled using custom CSS layered over Bootstrap components to ensure clarity and aesthetic consistency. Where necessary, JavaScript is used to dynamically load structural content and handle edge cases, such as missing or malformed PDB files.

### 2.4 Deployment and Hosting

The aptamer web server is deployed on a secure local Linux-based server managed by the development team. The application is served using Gunicorn WSGI^48^ as the Python Web Server Gateway Interface, which handles the communication between the Django backend and the web server. For high-performance request handling and static file delivery, Nginx^49^ is used as a reverse proxy. This configuration allows efficient serving of both dynamic and static content, including large PDB files.

The web server is containerized for ease of management and is continuously monitored to ensure high availability. File uploads, including 3D structures in PDB format, are stored in structured directories with controlled access to maintain data integrity. The server is hosted within the institution’s internal network, with controlled user access and administrative oversight to ensure security and long-term maintenance.

## 3 Usage Guidelines

The user guidelines are also outlined on the database homepage, providing a clear overview of the platform’s main functions. From the homepage, users can access the Visualize Aptamers tool to explore the data set interactively, download aptamers in JSON format for computational analysis, or submit new aptamer entries pending administrator approval. The JSON data includes detailed sequence information, molecular descriptors, secondary structure features, and minimum free energy (MFE) values, making it highly suitable for downstream machine learning applications such as classification, clustering, or regression modeling. An Admin Login option is available for database management and content approval. For structural data, users can download all PDB files in a compressed ZIP file, and the Search by Sequence function enables quick retrieval of aptamers matching a specific nucleotide sequence. This workflow and feature organization can be seen in Figure 1, as shown below.

**Figure 1:**
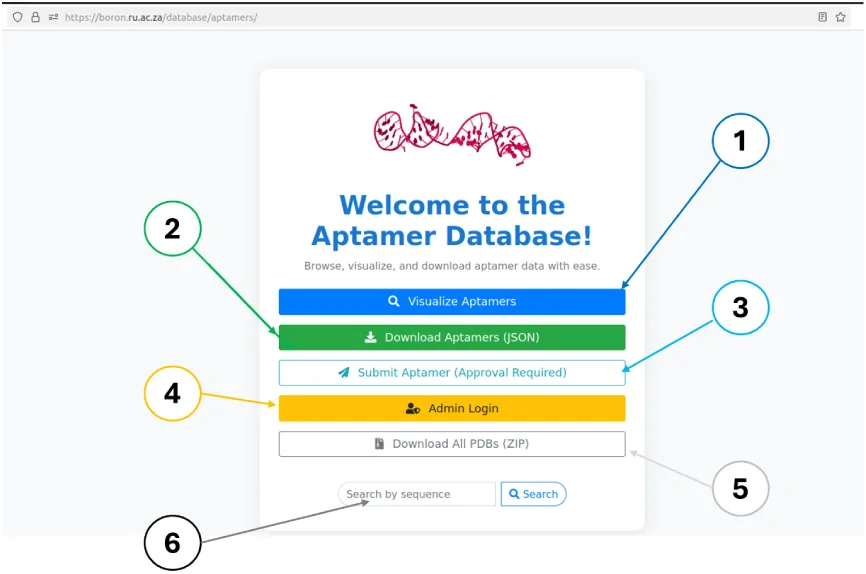
A home web interface for the Aptamer Database.

When the “Visualise Aptamers” button is clicked for an aptamer, the system redirects you to a dedicated page displaying detailed information about each aptamer as shown in Figure 2. Figure 2 shows the interactive view available for each aptamer entry in the database. From this interface, users can either download individual aptamer data in JSON format or visualize the aptamer directly within the platform. The visualization mode launches an embedded JSmol^47^ viewer, or VARNA^46^ allowing users to rotate, zoom, and inspect the aptamer structure in detail. Alongside the 3D model, the 2D structure can also be visualized. The 3D model should nevertheless be regarded as computationally predicted models rather than experimentally validated structures, the refinement and minimization performed by RNAComposer^41^ improve the structural geometry of the generated models but do not establish that the predicted conformations reproduce the experimentally adopted structures in solution.

**Figure 2:**
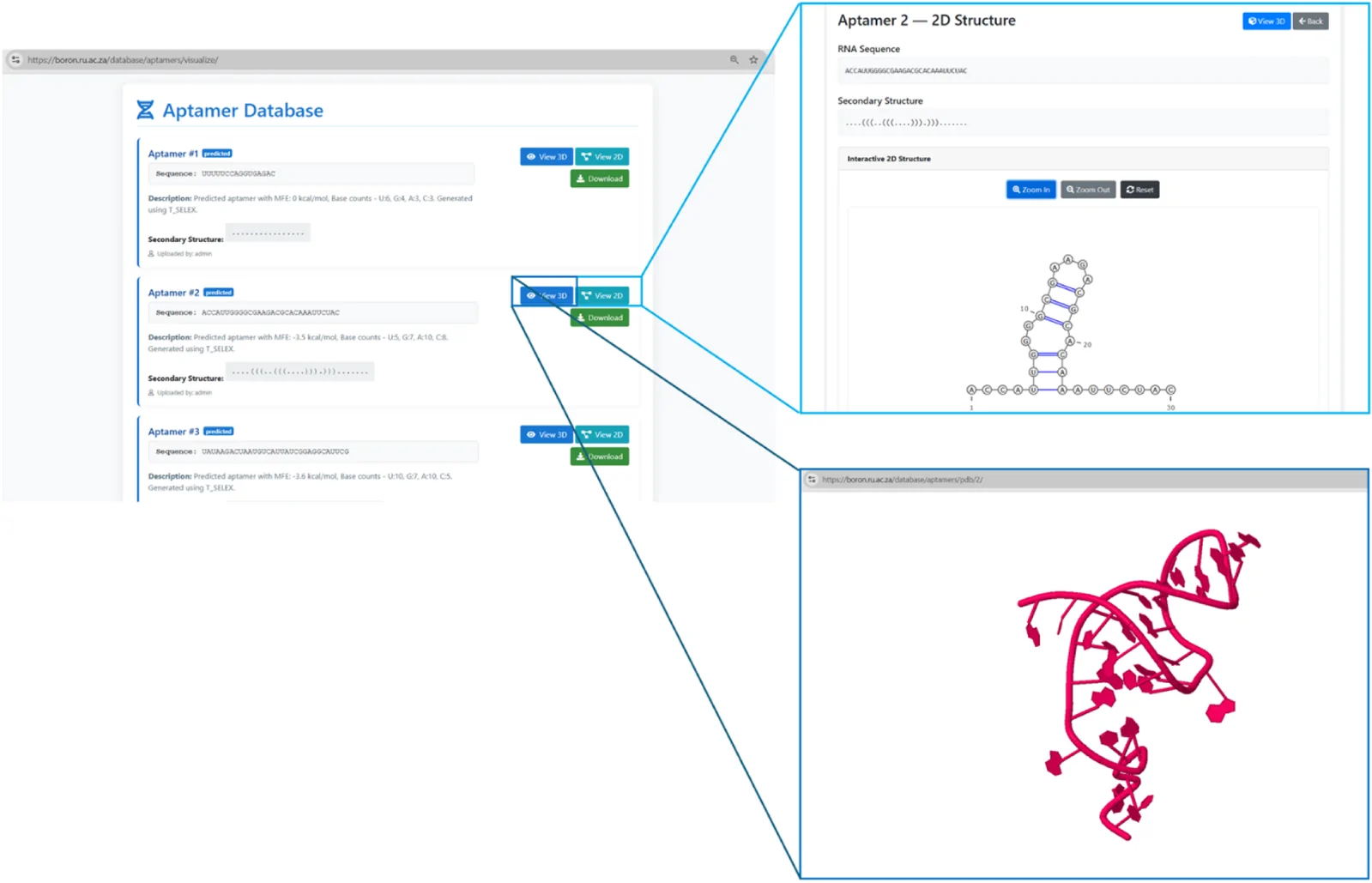
Web interface for interactively viewing and downloading the individual aptamer 3D structure and further visualizing the secondary structure.

As shown in Figure 3, clicking the search button on the homepage (Figure 1) without entering any query directs the user to the search results interface. This page provides both basic and advanced search functionalities. In the advanced search mode, users can filter aptamers based on a specified minimum and maximum MFE range, search for sequences containing specific nucleotide patterns, or locate an aptamer directly using its unique database number if known. The search results display each matching aptamer along with its sequence, description, predicted MFE structure, and options to either visualize the 3D structure or download the corresponding PDB file. Once the desired aptamers have been filtered, the platform allows users to download them in JSON format and corresponding 3D structures as a ZIP file, enabling convenient offline analysis and storage.

**Figure 3:**
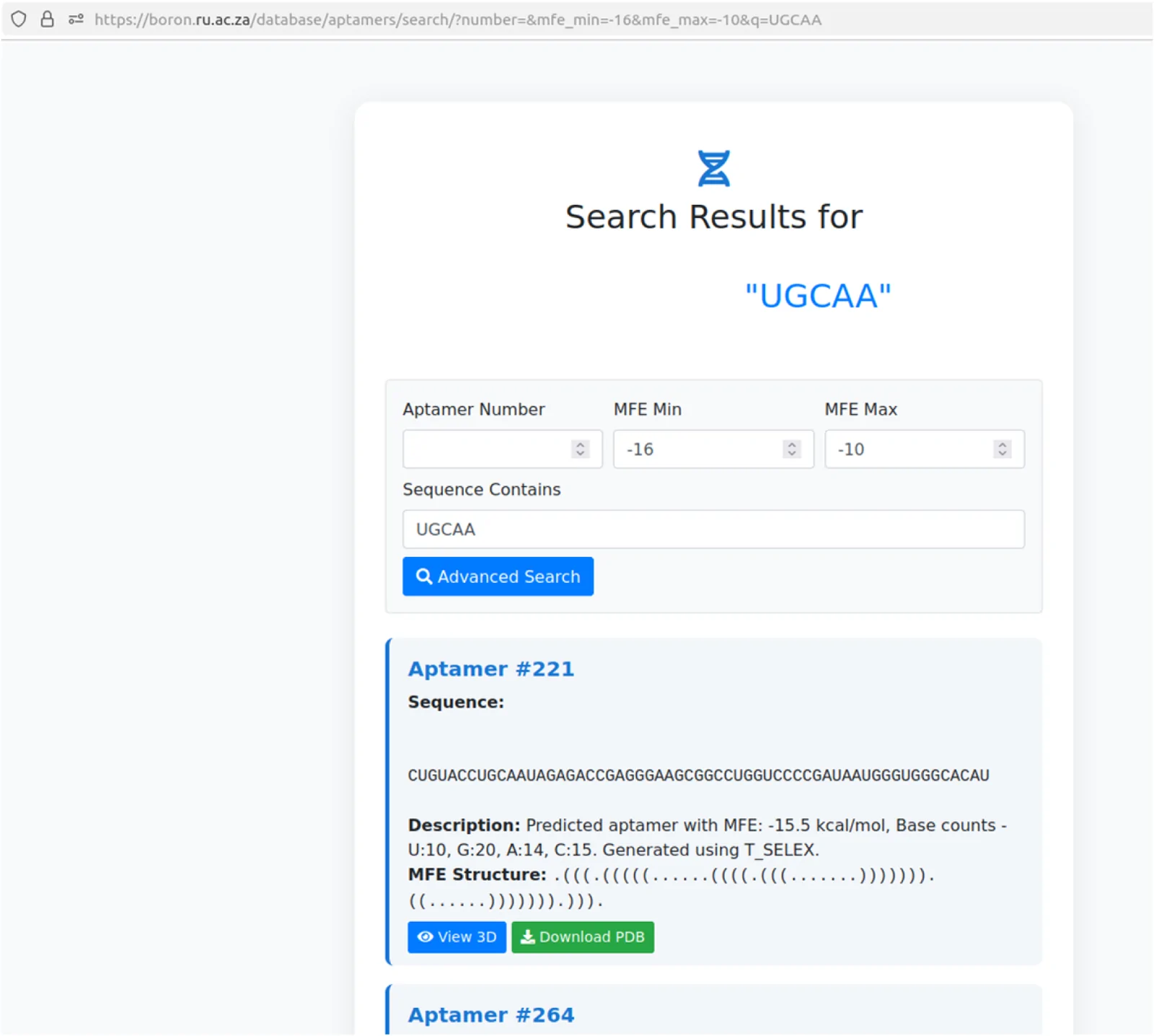
Advance search section together with filter settings.

In our database, we welcome growth through the active submission of new aptamers, as shown in Figure 4. The submission interface is designed to ensure that all contributed entries meet consistent quality standards. Each aptamer record begins with an automatically generated entry number, followed by required fields for the nucleotide sequence and a concise description. Contributors must classify the datatype of the aptamer as either *predicted* or *experimental*; for experimental entries, a peer-reviewed literature reference is mandatory to validate the data. Structural details, including secondary structure and minimum free energy (MFE) values, must be provided, with tools such as RNAfold recommended for MFE calculation. The submission form also captures data type for systematic categorization, as well as uploader details (name and e-mail) to maintain attribution and enable follow-up.

**Figure 4:**
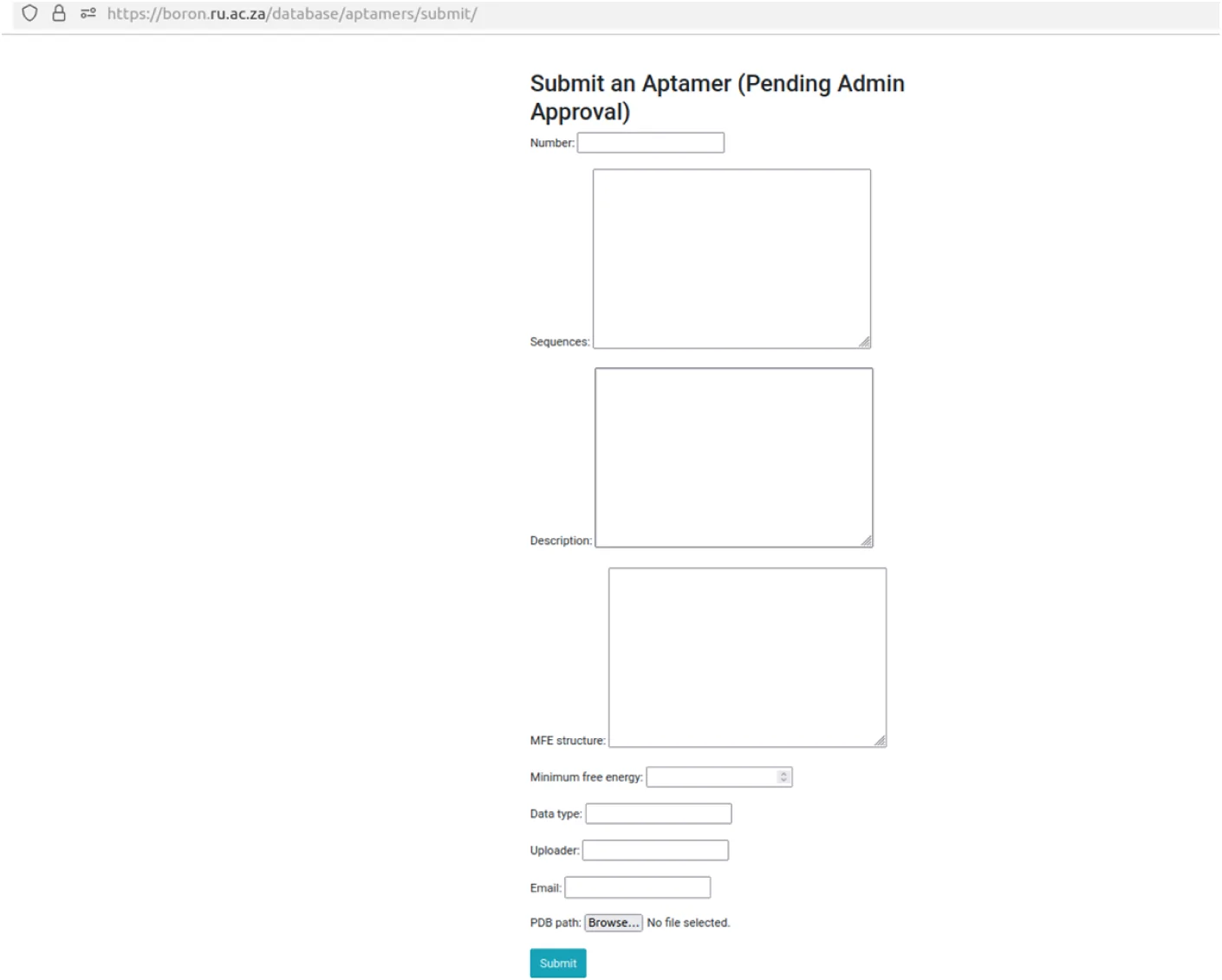
Web interface for uploading aptamers to the aptamer to the aptamer database.

## 4 Data Set Characterization and Limitations

### 4.1 APDB Characterization

To characterize the aptamer data set, we applied two complementary dimensionality reduction techniques: Principal Component Analysis (PCA) and t-distributed Stochastic Neighbor Embedding (t-SNE). About 161 features representing diverse molecular properties of the aptamers. These features encompassed minimum free energy (MFE), secondary structural patterns, stem density, GC- and AU-pairing ratios, sequence complexity, specific pairing paradigms, loop structures, and characteristic motifs.

The PCA results (Figure 5) revealed a clear separation of aptamers according to their structural and thermodynamic profiles. A notable trend was observed in the distribution of MFE values: most aptamers displayed relatively high MFE, clustering within the range of 0 to −5 kcal/mol, primarily located in the yellow regions toward the right-hand side of the PCA plot. Distinct clusters emerged for lower MFE ranges, including a green-shaded cluster corresponding to −10 to −20 kcal/mol, and a much smaller, dark-shaded group of aptamers with extremely low MFE values, around −30 kcal/mol. Interestingly, aptamers positioned below −5 along PC2 tended to exhibit a high AU-pairing composition, suggesting a relationship between base-pairing patterns and their projection in PCA space.

**Figure 5:**
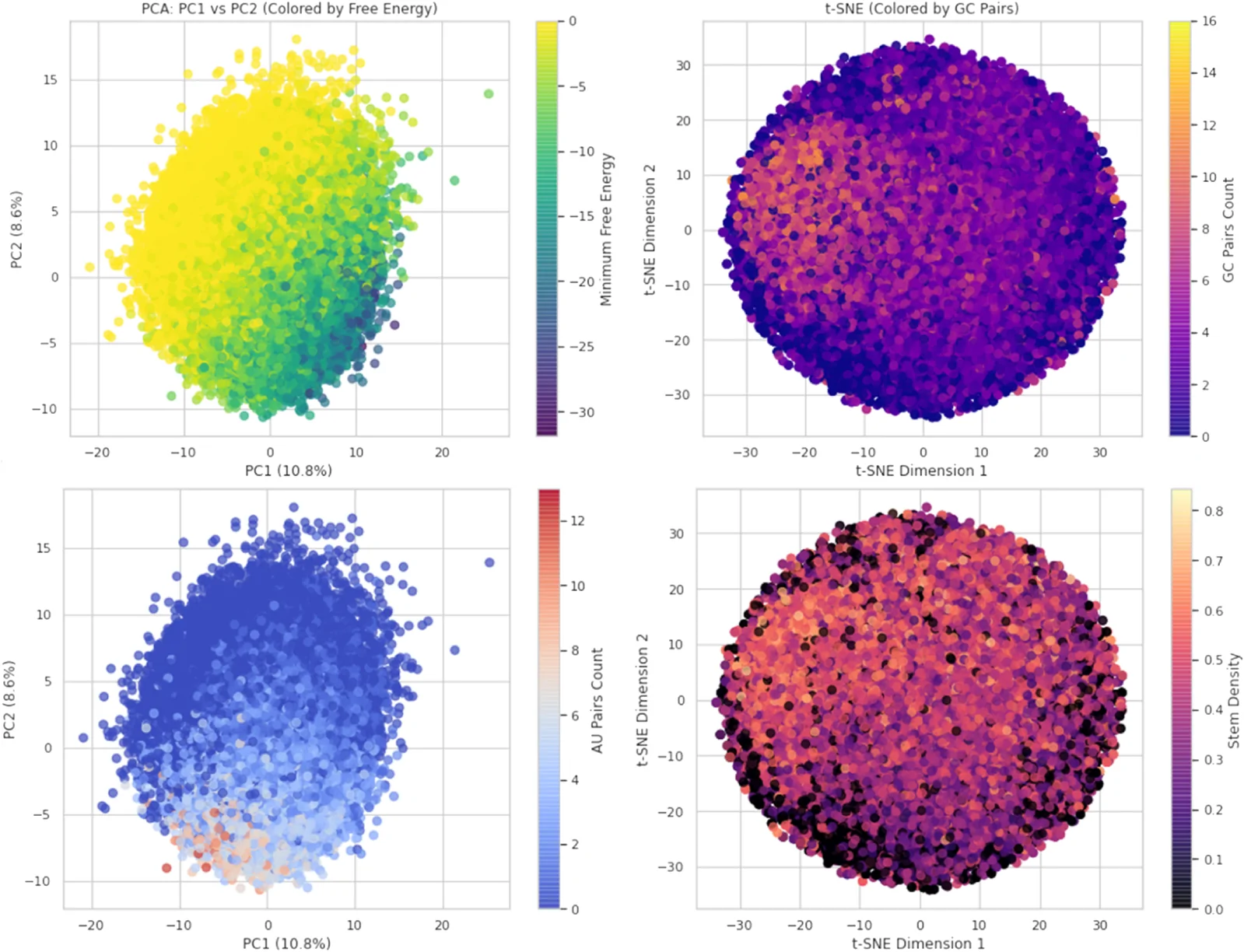
Dimensionality reduction of aptamer sequence structures using PCA and t-SNE.

In parallel, the t-SNE analysis provided a more nuanced view of local clustering patterns, highlighting compositional and structural affinities that may not be evident in the linear PCA space. One distinct observation was a dense, round cluster of aptamers with high GC-pairing content up to 20% occupying the central region of the t-SNE plot. Within this cluster, an orange-toned subset on the left-hand side indicated aptamers with especially high GC-pairing levels. Additionally, t-SNE revealed that many aptamers exhibited high stem density, suggesting a tendency toward stable, well-organized secondary structures. This reinforces the notion that GC-pairing and stem density are major contributors to aptamer stability and could be important determinants of their functional properties.

To visualize how different base-pairing compositions influence aptamer folding, we generated a 3D surface plot using GC-pair count, AU-pair count, and minimum free energy (MFE) as the three axes (Figure 6). Since folding stability is measured by MFE, this visualization provides a direct way to examine the relationship between pairing composition and thermodynamic stability. The resulting surface is not smooth; instead, it resembles a rugged landscape of hills and valleys. Aptamers with very low GC and AU pairing appear on the higher “hilltops,” while increasing either GC or AU pairing generally leads to a downward slope in the surface, reflecting lower MFE values suggesting more stable folded aptamers. However, the surface is not perfectly regular at certain points, the downward trend is interrupted by irregular “bumps” and plateaus. These suggest that folding stability does not always change uniformly with pairing composition, and that certain combinations of GC and AU content produce unexpected stability patterns, possibly due to complex secondary structure arrangements or sequence-specific interactions. Overall, the surface indicates that increasing GC pairing strongly reduces the minimum free energy, suggesting greater structural stability of the aptamers. Even when AU pairs are relatively few, aptamers with higher GC pairing can still achieve stable folding, whereas sequences dominated by AU pairs without sufficient GC pairing tend to exhibit less stable (or higher) MFE values.

**Figure 6:**
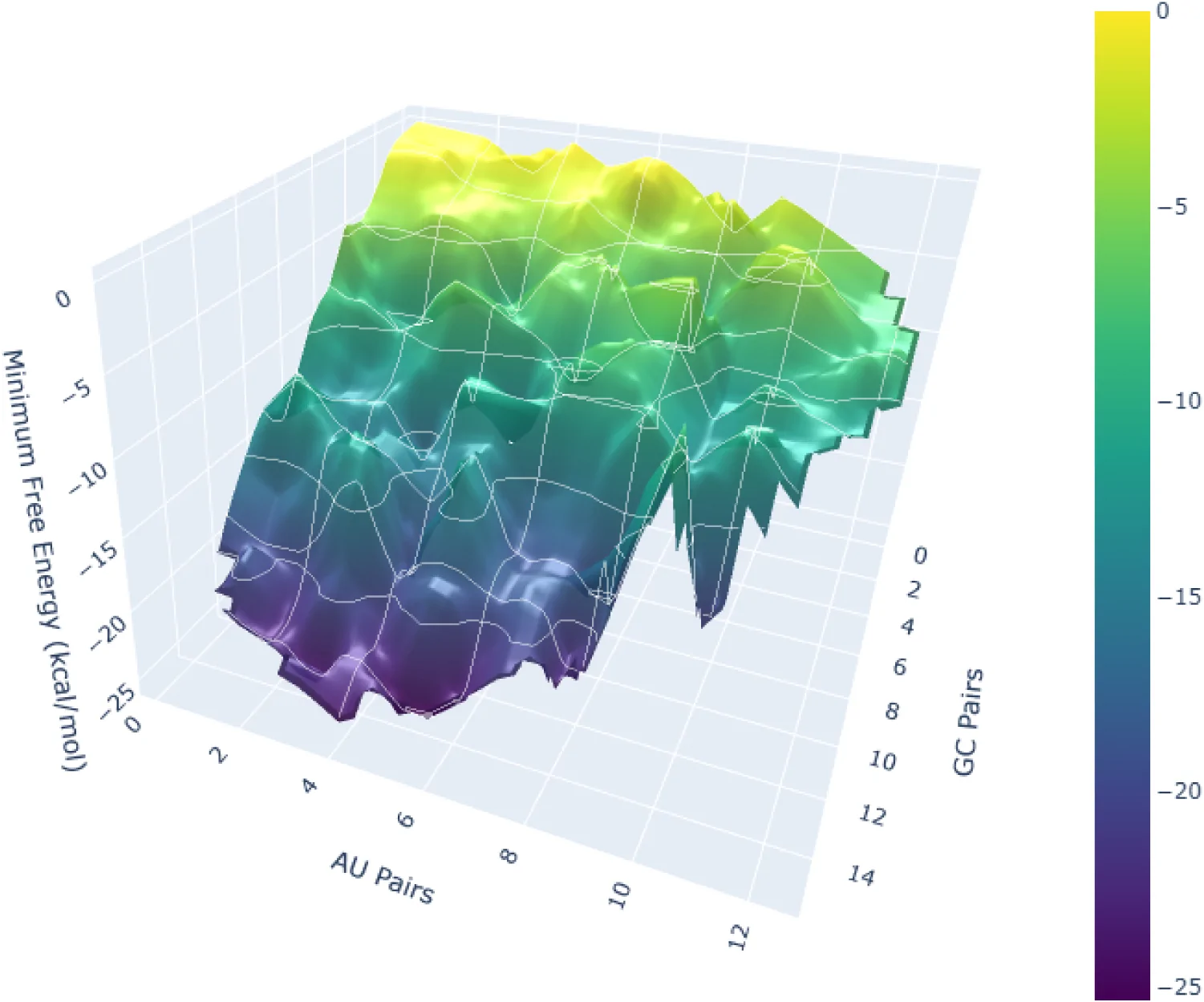
3D surface plot showing the relationship between GC pairing, AU pairing, and minimum free energy in aptamer folding.

Figure 7 presents two complementary sequence logo visualizations that describe the compositional and thermodynamic characteristics of the RNA aptamer data set. The upper panel shows a position frequency logo derived from a position frequency matrix (PFM) constructed from all aptamer sequences. At each sequence position, the occurrences of nucleotides (A, U, G, and C) were counted and converted into probabilities, allowing the relative nucleotide frequency to be visualized across the alignment. The height of each letter therefore represents the probability of observing a specific nucleotide at that position in the data set. This representation highlights the overall sequence variability of the aptamers, indicating that although certain positions may show enrichment of particular nucleotides, the data set largely maintains a heterogeneous distribution. Such diversity reflects the flexibility of RNA aptamers to adopt different sequence patterns while still forming functional secondary structures capable of binding molecular targets.

**Figure 7:**
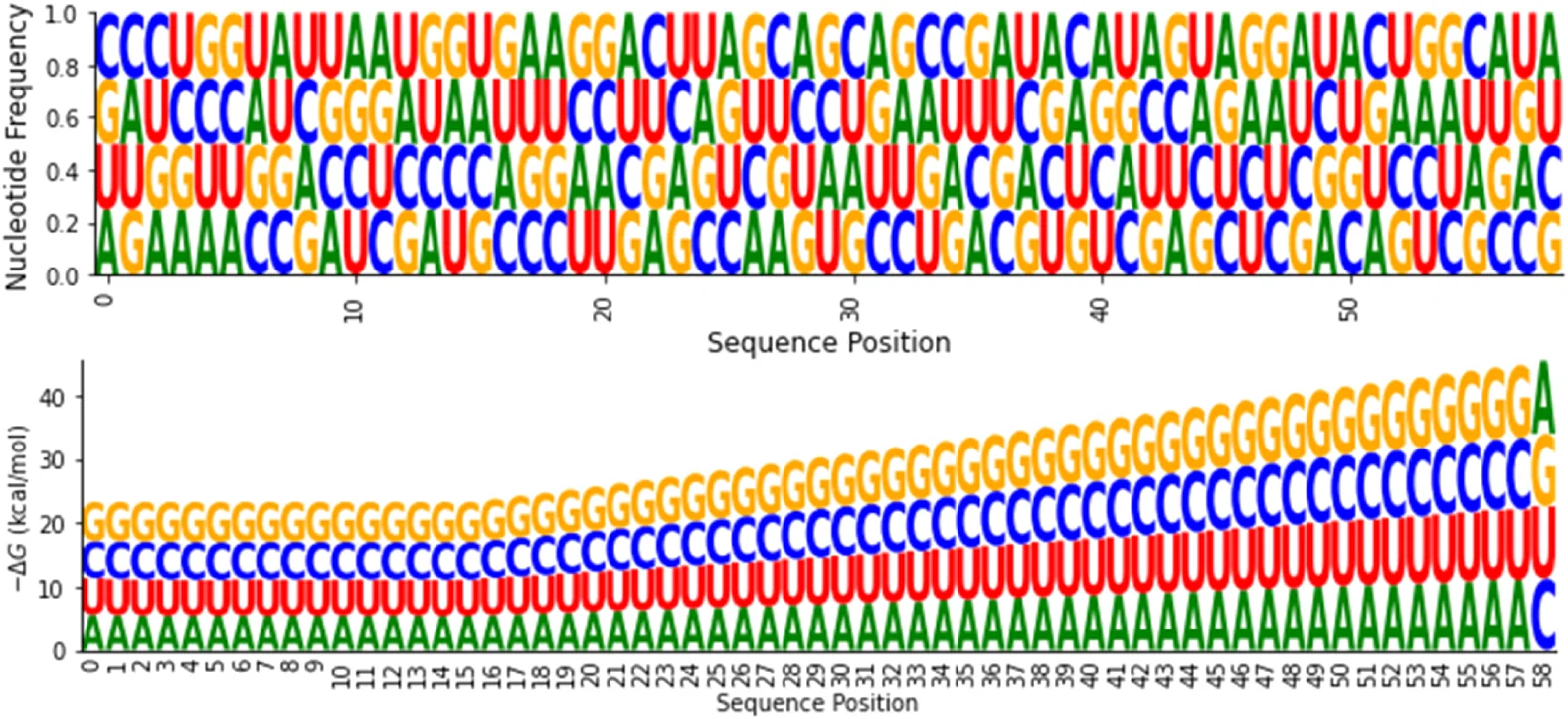
Dimensionality reduction of aptamer sequence structures using PCA and t-SNE.

The lower panel incorporates thermodynamic information by weighting nucleotide contributions using the negative Gibbs free energy (−ΔG) derived from the predicted minimum free energy (MFE) values of the aptamers. In this analysis, the average MFE associated with each nucleotide at each sequence position was calculated and multiplied by −1 so that larger values correspond to stronger stabilizing contributions to RNA folding. Because RNA stability increases as ΔG becomes more negative, nucleotides associated with more stable conformations appear taller in the energy logo. The visualization reveals that G and C nucleotides tend to contribute more strongly to lowering Gibbs free energy, consistent with the higher thermodynamic stability of GC base pairs, which form three hydrogen bonds and exhibit stronger stacking interactions. In contrast, A and U nucleotides generally show smaller −ΔG contributions, reflecting the weaker stability of AU pairs. Together, these two panels illustrate both the sequence diversity and thermodynamic drivers of aptamer folding, showing how nucleotide composition influences structural stability within the data set.

### 4.2 Limitations and Considerations for Machine-Learning and Other Applications

Although the size and structural annotations of APDB make it potentially valuable for downstream machine-learning applications, the data set should not be regarded as an experimentally validated ground-truth data set. The secondary structures and associated MFE values were generated using RNAfold,^38^ while the corresponding three-dimensional structures were subsequently generated using RNAComposer.^41^ Consequently, APDB may inherit method-specific limitations and systematic biases from both prediction procedures. RNA secondary-structure prediction is based on thermodynamic models and simplifying assumptions that cannot fully capture the conformational complexity of RNA, and predicted structures may therefore differ from experimentally observed conformations. The ViennaRNA developers also acknowledge limitations in the underlying thermodynamic parameters and note that experimental probing information can improve secondary-structure prediction accuracy.^50^

RNAComposer provides a well-established and competitive approach for RNA three-dimensional structure prediction and offers practical advantages over computationally intensive physics-based approaches. Nevertheless, its predictions remain subject to limitations.^51^ As a knowledge- and fragment-based method, RNAComposer depends on the availability and suitability of structural fragments and on the accuracy of the supplied secondary structure.^41^ Consequently, limitations or inaccuracies in the predicted secondary structure can propagate into the resulting three-dimensional model. Recent assessments of RNA 3D structure prediction have shown that template- and fragment-based approaches, including RNAComposer, can be constrained by the structural diversity of available templates and by the accuracy of the input secondary structure.^51,52^ Moreover, the emergence of machine-learning and deep-learning approaches has improved RNA 3D structure prediction in several settings, but these methods are themselves subject to limitations, including dependence on experimentally determined training structures, incomplete representation of RNA structural diversity, and difficulties in generalizing to structures outside their training distributions.^52^ Thus, neither established computational approaches nor newer machine-learning methods should be considered universally reliable substitutes for experimental structural determination. Computationally generated RNA 3D models may still contain structural imperfections, including steric clashes, geometric anomalies, and irregularities in local or global topology, even following computational refinement.^53^ These limitations should therefore be considered when interpreting or using the predicted 3D structures in APDB, particularly for applications requiring high structural accuracy.

These considerations are particularly relevant because structural prediction errors can propagate through the APDB generation pipeline. Previous studies have shown that relatively small changes in predicted secondary-structure constraints can result in substantial differences in the resulting three-dimensional models, while computationally generated RNA structures may also exhibit steric clashes, abnormal geometries, or topological irregularities.^54–56^ Accordingly, machine-learning models trained on APDB should be interpreted with appropriate caution, as they may learn not only biologically meaningful relationships but also characteristics and biases associated with the underlying RNAfold and RNAComposer prediction procedures. High predictive performance on APDB alone should therefore not be interpreted as evidence of experimental predictive accuracy or biological generalizability.

Rather, APDB is intended as a large computational resource for hypothesis generation, method development, computational aptamer design, and preliminary machine-learning investigations. Its value for these applications should be considered in conjunction with its computational origin. Models developed using APDB should, where possible, be evaluated against independent experimentally characterized RNA data sets to determine whether relationships learned from the computationally generated data transfer to experimentally observed structures and thermodynamic properties. Future development of APDB will therefore focus on integrating experimentally determined structures, experimentally measured thermodynamic and structure-probing data, and systematic structural-quality assessment of the predicted three-dimensional models.

## 5 Conclusion

The Aptamer Database (APDB) consolidates over 150,000 computationally generated RNA aptamers annotated with sequences, predicted secondary structures, 3D structures and MFE, providing an integrated platform for exploration, visualization, and data export. By combining structural annotations with interactive tools, APDB fills a critical gap in current repositories and supports both fundamental research and machine learning driven design. Our data set analysis using PCA, t-SNE, and 3D surface plots revealed distinct compositional patterns, with GC and AU pairing ratios showing strong influence on folding stability, as reflected in minimum free energy values. The observed surface topography suggests nonlinear stability changes as pairing ratios vary, offering insights for rational aptamer optimization. APDB’s modular architecture ensures scalability for incorporating DNA aptamers, experimental data sets, and aptamer–target complexes, ultimately enhancing applications in drug discovery, biosensing, and computational modeling.

## Data Availability

The database is freely available at the Web site https://boron.ru.ac.za/database.
